# A Deep Neural Network-Based Method for Prediction of Dementia Using Big Data

**DOI:** 10.3390/ijerph18105386

**Published:** 2021-05-18

**Authors:** Jungyoon Kim, Jihye Lim

**Affiliations:** 1 Department of Computer Science, Kent State University, Kent, OH 44242, USA; jykim2@kent.edu; 2 Department of Health Care and Science, Donga University, Nakdong-Daero 550 beongil 37, Saha-Gu, Busan 49315, Korea

**Keywords:** deep learning, deep neural network, dementia, feature extraction, prediction, principal component analysis

## Abstract

The rise in dementia among the aging Korean population will quickly create a financial burden on society, but timely recognition of early warning for dementia and proper responses to the occurrence of dementia can enhance medical treatment. Health behavior and medical service usage data are relatively more accessible than clinical data, and a prescreening tool with easily accessible data could be a good solution for dementia-related problems. In this paper, we apply a deep neural network (DNN) to prediction of dementia using health behavior and medical service usage data, using data from 7031 subjects aged over 65 collected from the Korea National Health and Nutrition Examination Survey (KNHANES) in 2001 and 2005. In the proposed model, principal component analysis (PCA) featuring and min/max scaling are used to preprocess and extract relevant background features. We compared our proposed methodology, a DNN/scaled PCA, with five well-known machine learning algorithms. The proposed methodology shows 85.5% of the area under the curve (AUC), a better result than that using other algorithms. The proposed early prescreening method for possible dementia can be used by both patients and doctors.

## 1. Introduction

Dementia is closely related globally to elderly disability and dependency. Nearly 50 million people suffer from dementia, and about 10 million new patients appear yearly [1]. Physical, psychological, social, and economic aspects of dementia affect a diverse group of people, including individuals with dementia as well as their caregivers, family members, and society in general. According to World Alzheimer’s report in 2015 [2], the prevalence rate of dementia in 2015 in the Asia Pacific High Income demographic, which includes South Korea, was about 7%. The increased rate of dementia patients is also expected to reach about 56% between 2015 and 2030 [1]. The increasing prevalence of dementia patients in Korea has caused treatment costs and social burdens for dementia patients to significantly increase. The National Assembly Budget Office has asserted that the social costs of dementia will increase from 11.7 trillion won in 2013 to 23.1 trillion won in 2030 and 34.2 trillion won in 2040 [3]. Despite the importance of establishing dementia policy, epidemiological studies related to dementia are deficient, creating a great need for related research.

The primary signs and symptoms of dementia include memory loss, difficulty with tasks, disorientation, language problems, behavioral changes, and loss of initiative. Signs and symptoms related to dementia occur in three stages, i.e., early stage, middle stage, and late stage. The early stage is somewhat ambiguous due to the disease’s gradual progress; it includes events such as losing track of time, forgetfulness, and becoming lost in familiar places. The signs and symptoms of the middle stage are clearer than those of the early stage; people in the middle stage sometimes become lost at home and forgetful of current events and names. Difficulty with communication and increasing need for personal care are other symptoms. Behaviors are changed, with repeated questioning and wandering. The late stage reflects abnormal symptoms with virtually total dependence and inactivity due to serious memory disturbances. Detailed symptoms and signs include difficulties in walking, radical behavioral changes, failures at recognizing time and place, and failures in recognizing relatives and friends. These and other signs and symptoms represent differences among the stages associated with the progress of dementia [1].

The general diagnosis process of dementia requires comprehensive evaluation such as listening to medical history, cognitive function and mental state evaluation, neuropsychological testing, evaluation of daily-living activities, clinical laboratory testing, and brain imaging testing [1,3]. During the first stage, clinicians evaluate cognitive function and mental state based on the Mini-Mental State Examination (MMSE) tool [4]. During the second stage, the Korean version of the Consortium to Establish a Registry for Alzheimer’s Disease (CERAD-K) is used as a neuropsychological test for comprehensively assessing the state of cognitive functionality of dementia patients [5]. During the final stage, magnetic resonance imaging (MRI) or computed tomography (CT) and hospital-based blood tests are used to diagnose patients. These imaging techniques are simple tools that can also be used to highlight morphology changes and irregularities, with MRI and CT scans mostly used to distinguish the biomarkers of neurodegenerative diseases [6]. The test results permit placement of suspected patients into dementia, mildly cognitive-impaired (MCI), and normal categories [7].

There have been many recent efforts based on big data analysis to extend precision in many medical areas [8,9,10]. Precision medicine can be loosely defined as patient-centric therapy and diagnosis [11]. Improving prognostic models based on electronic health records (EHRs) and healthcare claim data [12] can be used to support precision medicine. Big data analysis associated with a deep learning method has been used to predict health status or disease [13,14,15], and deep learning has recently been widely applied in many areas, with various satisfactory results reported where previous conventional solutions have been inadequate [16,17]. Xu et al. (2017) used the deep learning model to achieve more solid and generally better model performance and least absolute shrinkage than for a selection operator (LASSO) model, a generalized linear model (GLM), and an autoregressive integrated moving average (ARIMA) model [18]. Most image data produced by deep learning as supervised learning have been annotated by well-trained experts [19,20]. The performance of image-based deep learning models may possibly depend on the training and experience of the involved radiologist. Although deep learning has been used to predict occurrence of diverse diseases, few studies have attempted to predict dementia based on big non-image data analysis.

In this paper, we examine factors affecting dementia incidence and develop a predictive model based on scaled principal component analysis (PCA) and DNN, employing the 2001 and 2005 Korea National Health and Nutrition Examination Survey (KNHANES) datasets [21]. The proposed methodology specifically uses the indirect and limited number of features from this easily accessible data to predict dementia. The proposed methodology will provide appropriate information to healthcare policymakers for improving the quality of medical care and evaluating its appropriateness, and will improve efficiency in use of diagnostic resources. The proposed methodology was validated for potential dementia prediction using massive EHRs, and we expect to expand the proposed method for prescreening various other health issues in the e-health field.

## 2. Related Work

There have been numerous studies using deep learning approaches to resolve issues in diverse areas [22,23,24,25,26], and deep learning methods for detecting many diseases support the further development of computer-aided diagnosis systems [27,28,29]. The detection or prediction performance of deep learning/machine learning on different diseases has been verified using diverse approaches and datasets

Previous heuristic or nature-inspired methods for detecting dementia and some deep learning methodologies using imaging data have been reported. Morales et al. [30] applied several machine learning models such as the support vector machine (SVM) and different types of naïve Bayes to predict dementia. That methodology used 112 variables obtained from MRIs of 45 patients (14 patients with dementia). Although the prediction accuracy was high (96%), the dataset sizes were relatively small. Korolev et al. [31]. proposed a prognostic prediction model related to MCI-to-dementia progression using MRI data. Most recently, Battineni et al. [32] applied the support vector machine (SVM) using a long-term collection of 373 MRI data from 150 subjects in the Open Access Series of Imaging Studies (OASIS-2). They categorized the dataset in terms of clinical dementia ratio scores, viz., non-demented (190), demented (146), and converted (37), and the accuracy and precision were respectively 68.75% and 64.18%. This study used a relatively low number of subjects and exhibited low prediction performance. Frolich et al. [33] used a bootstrapping wrapper around an SVM and a linear kernel based on the dataset of the MCI patients. From among the 1071 MCI patients, they selected a subsample of 115 patients who had progressed to dementia, and an AUC value up to 0.83 was shown. They concluded that using two biomarkers of neurodegeneration was no better than using a single parameter for diagnosing the progressed dementia from MCI. Zhou et al. [34] applied a stage-wise deep neural network (DNN) to diagnose dementia through feature-learning methodology using neuroimaging data. The subject ratio of actual positive and negative dementia was 190 and 226, and the normal and dementia accuracies were 60.8% and 58.7%, respectively. Machine learning-based approaches using image data may also be limited as screening tools because of the subtle atrophy during early stages of the disease and overlap in atrophy patterns between dementia types. 

There have been a few recent studies regarding dementia prediction based on statistical data. So et al. [35] provided a dementia-detection system using several machine learning techniques, using data consisting of 9799 in the normal group and 4201 in the cognitive-decline group. F-measure values of normal based on multi-layered perceptron and of dementia based on support vector machine were 0.97 and 0.73, and although the overall detection performance of the study was relatively accurate, their method used mental state data from relatively sparse medical records. Their method divided the detection procedure into two phases. The first screening was accurate, but the second achieved relatively low performance. Barnes et al. [36] developed an electronic health record (EHR)-based tool to detect patients with unrecognized dementia. Among 16,655 records, 15,640 indicated no dementia, 498 indicated unrecognized dementia, and 517 indicated recognized dementia. They applied logistic regression with LASSO penalty to build a prediction model with an AUC value of 0.809. Although their discrimination was good, with a large population’s EHR, the generalizability was somewhat limited because the majority of participants were white, well-educated, and English-speaking. Park et al. [37] developed machine learning-based prediction models using health and healthcare history data to predict future incidence of Alzheimer’s disease. The total number of data points for elders of age greater than 65 years was 40,736 (614 dementia and 2026 probable AD data). They used multiple machine learning techniques in one-year prediction with AUC of up to 0.775, along with bootstrapping to make the data balanced and ensure that the diagnoses of AD in the database were not clinically ascertained. 

## 3. Subjects

**Figure 1:**
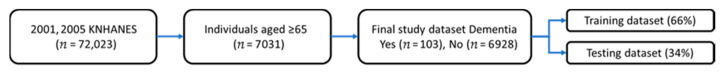
The data selection of the study population from KNHANES.

The materials used in this study were obtained from the 2001 and 2005 KNHANES, performed by the Korea Centers for Disease Control and Prevention (KCDC). The KNHANES was conducted nationwide as a cross-sectional study in accordance with Article 16 of the National Health Promotion Act. In KNHAES, participating households were randomly selected and sampled using multilevel stratification according to geographic area [38]. The KNHANES corresponds to research conducted by the government for public welfare in accordance with Article 2 of the Bioethics Act and is government-approval statistics based on Article 17 of the Statistical Act (Approval No. 117002). Researchers were allowed to use the data through the raw material use application procedure on the website of the Center for Disease Control and Prevention. Of the 72,023 participants who responded to KNHANES’ medical utilization and health behavior in 2001 and 2005, 7031 were adults over the age of 65. Based on previous studies and statistical indicators [39], we selected a population aged greater than 65. Of the subjects, the number of patients with dementia was 47 in 2001 and 56 in 2005. The presence of dementia, which is a dependent variable, was identified using the following question: “Have you had dementia for the past year?” or “Is there a limitation of activity due to dementia?” A total of 7031 subjects were finally included in this study. The dataset was divided into two parts: training (66%) and testing (34%) data, as shown in Figure 1.

## 4. Method

### 4.1. Overview

**Figure 2:**
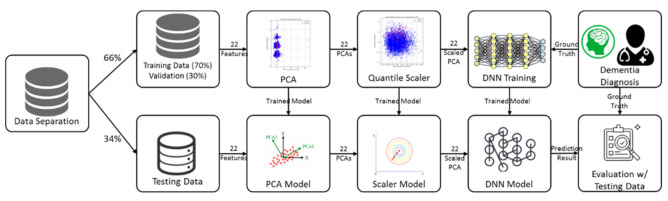
The Flow Chart of the proposed DNN/scaled PCA approach.

In the proposed methodology, 22 variables were used, such as year, gender, age, type of insurance, region (city/rural), marital status, education, the number of family members, household income, subjective health status, stress awareness, smoking status, the experience of drinking, regular exercise, and the presence or absence of comorbidity (diabetes mellitus, arthritis, hypertension, myocardial infarction, stroke, tuberculosis, asthma, and chronic renal failure). Among the 22 variables, based on previous studies [40,41], we selected age, sex, education, living place, insurance type, income, chronic disease, level of depression, drinking, smoking, and ADL. Other possible and indirect variables were used for testing the predictivity of the proposed model. In order to extract the numerical type of features from the raw input data, we applied a scaled PCA that is able to estimate the dementia-related risk factors. The dataset includes only 103 subjects with a history of dementia among the 7031 subjects. The developed system architecture is shown in Figure 2. First, the KNHANES data, for 22 input variables from 7031 subjects, was used to train (66%) and test (34%) models. In order to validate the training process, 30% of the training sample was used. Second, the categorical variables were converted to continuous variables based on a scaled PCA method as a preprocessing. Third, we trained the DNN model using the preprocessed variables and evaluated the predictive performances with the annotations labeled by clinicians. In order to evaluate the accurate testing results, the data for testing models were completely separated from the training data for testing.

### 4.2. Preprocessing

**Figure 3:**
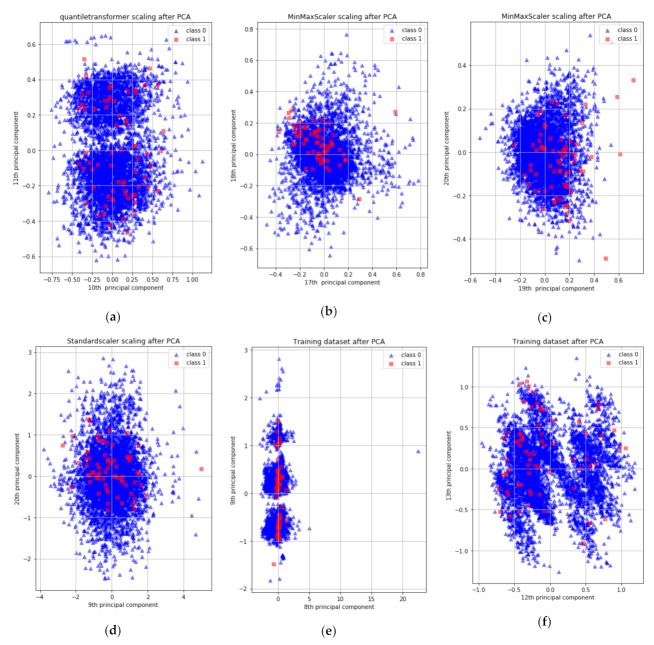
Diverse Feature scaling plots with the PCA: class 0 is non-dementia patients (blue triangle) and class 1 is dementia patients (red rectangular): (**a**) 10th and 11th PCA with quantile transformer scaler; (**b**) 17th and 18th PCAs with min/max scaler; (**c**) 19th and 20th PCAs with min/max Scaler; (**d**) 9th and 20th PCAs with standard scaler; (**e**) 8th and 9th PCAs without scaler; (**f**) 12th and 13th PCAs without scaler.

**Figure 4:**
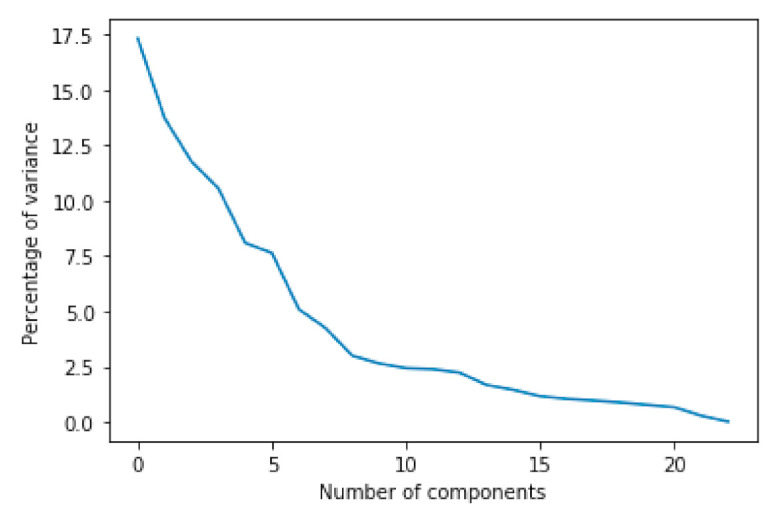
The percentage of variance in PCA-min/max-transformer scaler.

PCA is a method of converting the raw input data to new coordinate systems for extracting valuable information from complicated datasets [42]. In general, PCA is used to reduce the dimensions of the raw input data and find the optimal hidden characteristics for generating the preprocessed inputs of classification algorithms [43]. The used KNHANES dataset, however, consists of mostly categorical/binary and a small number of continuous variables with a lot of missing data. The categorical/binary variables need to be converted to continuous variables for increasing the overall detection performance due to a lack of specific information. We applied diverse scalers for enhancing the performance of PCA. The PCA with the scaler preprocessed all input data to generate new 22 variables to reduce the effect of the discrete data. The combinations of principal components (PCs) of six different PCAs are shown in Figure 3: (a) 10th and 11th PCs with quantile transformer scaler; (b, c) 17th, 18th, 19th, and 20th PCs with min/max scaler; (d) 9th and 20th PCs with standard scaler; (e, f) 8th, 9th, 12th, and 13th PCs without scaler. As can be seen in Figure 3b,c, the dementia data (red boxes) are gathered on one side, making it possible to see that the min/max method provides meaningful feature values compared to other PCA combinations. In addition, according to the testing results shown in Table 1, a DNN with PCA-min/max-transformer scaler shows the best mixture, and Figure 4 shows the percentage of variance.

### 4.3. DNN Architecture

**Figure 5:**
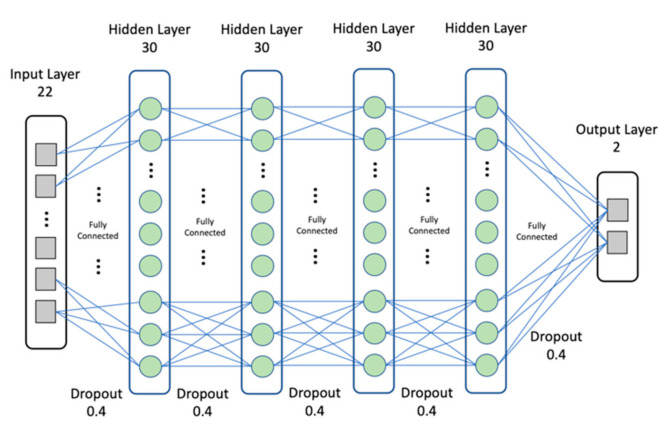
The architecture of the proposed DNN.

A simple feed-forward neural network was used to train the proposed model with a standard backpropagation algorithm. We have trained diverse combinations and optimized hyperparameters including the activation function, the regularization technique, the number of hidden layers, and the number of neurons in each layer. The network architecture of four hidden layers with each hidden layer containing 30 neurons showed the best performance. The last layer, with two neurons, generated a regression output. The ReLU activation [44] was applied in each hidden layer, sigmoid was applied in the output layer, and the dropout [45] probability was 0.4 for all hidden layers. Adam optimization [46] with the binary cross-entropy and 0.001 of the learning rate were used for the training process. We did not apply the weighted binary cross-entropy because increasing the weight of the minor classes’ losses may cause instability for optimizing performance in the highly imbalanced dataset [47]. The optimized hyperparameter choice is robust and has shown better predicting performance. Batch normalization [48] after the first three hidden layers and dropout were applied to avoid overfitting and unstable convergence. Figure 5 shows the detail of the DNN architecture.

We conducted comparative analysis using six classification algorithms: random forest (RF) [49], AdaBoost [50], multilayer perceptron (MLP) [51], Gaussian Naive Bayes (GNB) [52], SVM [53], and our proposed DNN with min/max scaler. All classifiers were evaluated using common performance metrics such as recall, specificity, precision, accuracy, receiver operating characteristic (ROC) curve, and area under ROC curve (AUC) using the KNHANES dataset. For a better comparison between the classifiers, we attempted to set the similar best-effort sensitivity and specificity results for all methods. Overall, performance can be compared using AUC value as a single performance metric to better reflect algorithm performance [54].

### 4.4. Performance Metric

In general, although the detection performance of the binary classifier was evaluated by accuracy (Acc), the KNHANES dataset is imbalanced in that there was much more non-dementia (*n* = 6928) data than dementia (*n* = 103) data. To evaluate the imbalanced data, three more metrics were used, viz., recall (Rc), specificity (Sp), and precision (Pc). Rc is the probability of predicting the subjects with dementia, Sp indicates the probability of detecting the subjects with non-dementia, and Pc reflects the probability of the algorithm’s correct classification of dementia status among the data classified as dementia. The four parameters, indicating positive or negative predictions based on true or false conditions, were used to screen status for the binary classifier. Mathematically, these performance metrics can be calculated using Equations (1)–(4).

Rc = TP/(TP + FN)(1)

Sp = TN/(TN + FP)(2)

Pc = TP/(TP + FP)(3)

Acc = (TP + TN)/(TP + FN + FP + TN)(4)

where the true-positive (TP) is correctly identified as dementia and the true-negative (TN) is correctly identified as non-dementia. The false-positive (FP) and false-negative (FN) are incorrectly identified status for dementia and non-dementia, respectively.

### 4.5. Hyperparameter Tuning for Optimal Result 

The training environment includes several hyperparameters that must be tuned for best-effort performance. We have tuned hyperparameters, including the DNN depth and the number of nodes, to build the optimal model and improve prediction performance. To the best of our knowledge, there is no general rule for tuning hyperparameters, so we established a system to train the depth of 2 to 8 layers and 10 to 50 nodes based on trial and error. To minimize the overfitting problem, we adopted two techniques, dropout and batch normalization. Dropout works as a weighting to prevent focusing on outcomes from specific hidden nodes, and batch normalization prevents the loss of feed-forward data on initialization in terms of appropriate weighting. We have trained the model based on dropout values ranging from 0.1 to 0.5 and, based on the testing, the optimal dropout value was chosen as 0.4. Testing results based on hyperparameter tuning are shown in Table 2.

## 5. Results and Discussion

We have tested various setting combinations and get the best DNN architecture for dementia prediction including four hidden layers, each with 30 neurons. The testing environment sets training epochs as 50 and a batch size as 10. We applied the preprocessed inputs of the scaled min/max PCA to the trained DNN model and obtained the confusion matrix, as shown in Table 3.

**Figure 6:**
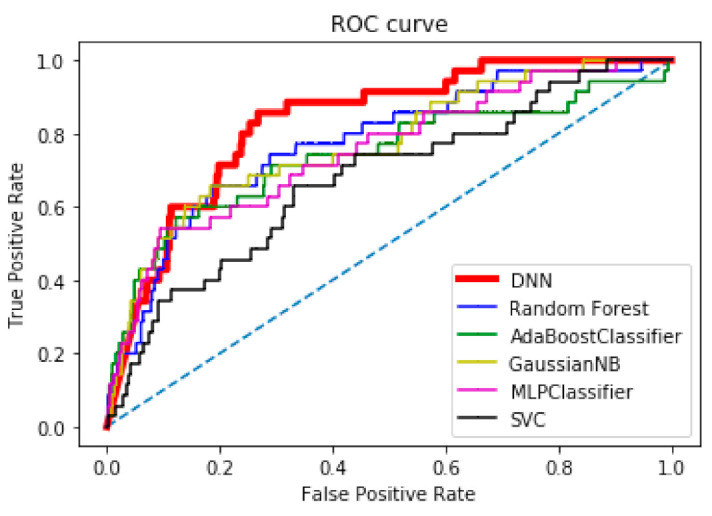
Comparison of ROC curves of six classification methods.

Table 4 summarizes the performance characteristics of the six classification algorithms for classifying subjects as having dementia for the given testing data. The optimal threshold for classifying dementia was 0.025, based on all model parameters, resulting in an Acc of 81.9%, an Rc of 68.6%, and an Sp of 82.1%, as marked italic. The AUC value represents the overall performance as one value; the top two AUC values are highlighted in bold. All thresholds of the compared classifiers have been adjusted to balance values of sensitivity and specificity. Figure 6 compares the six classifiers using ROC curves with the best ROC curve marked as a bold red line. The proposed scaled PCA/DNN method produced the best result, followed by the RF method. Based on comparative results, we conclude that the scaled PCA/DNN method outperforms the other classifiers in terms of all performance metrics. We also compared the proposed method with other studies, as shown in Table 5. Although previous studies used different statistical features with different conditions, such as the ratio of normal and dementia and performance metrics, the proposed method produced the best AUC result (0.855) compared to the others’ AUC values, except for [37] that used a highly balanced dataset. 

Table 6 shows the derived correlation coefficients of the 22 input variables with respect to dementia; the best correlation (greater than 0.14) is marked in bold and the next three informative values (over ±0.07) are marked in red italics. Based on Table 3, age is the most correlated input variable, with the next three factors being the number of family members, stroke, and subjective health status. The data in this study are basically a type of subjective health awareness that reflects a self-reported assessment of one’s own health status. While the survey of subjective health perception of respondents is relatively simple, the survey of actual health status is much more difficult. Although the questionnaires of subjective health perception based on the Likert scale of 4 or 5 are the variables that contained possible measurement errors, subjective health perception has been widely used in social science research as a proxy variable for actual health status. We considered not only the individual subjective health status, but also the interconnected relationships based on the proposed scaled PCA and DNN. Thus, although the individual correlation coefficient of the 22 variables in Table 6 is relatively low, the overall AUC of the proposed methodology shows high performance. 

The proposed methodology was able to predict the dementia population, based on limited or indirect data such as health behavior and medical service usage records. It could be an initial screening tool to facilitate diagnosis or reduce medical costs. The limitations of this study are the use of a seriously imbalanced dataset and a lack of longitudinal data reflecting the progress of dementia. From our previous research, the proposed methodology has powerful potential for using big data to predict diverse health-related problems such as stroke [55] and osteoarthritis [56], and we expect that a balanced dataset could resolve problems of the high false-positive rate and low precision. The survey data also include binary or categorical information, and although we used PCA preprocessing to convert discrete data to continuous form for improving resolution, additional input variables were still required. The dataset we used was also targeted to subjects who possibly might suffer from dementia in the near future, and subjects currently under medical treatment for dementia were excluded. This pre-selection process could impact the overall predictive performance of the proposed model.

## 6. Conclusions

In this paper, we have proposed an automatic dementia-prediction methodology that uses a combination of a PCA with min/max transforming scaler and a DNN with 7031 subjects from a health behavior and medical utilization record dataset. No subjective inputs were used in the proposed methodology. The proposed model can be used for early detection of potential dementia patients who might need additional medical checkups and treatment at the appropriate time before disease exacerbation. Because of unsupervised clustering, the proposed scaled min/max PCA does not require manual variable selection. Because input data were relatively simple, DNN was applied to examine significant variables and scaled min/max PCA values to extract features as continuous variables from discrete/categorical input variables. The Rc, Sp, and AUC values resulting from the proposed method were 68.6%, 82.1%, and 85.5%, respectively. The proposed methodology predicts not only future dementia patients but also other types of diseases using data that include limited input variables. 

Future studies should examine the analysis of other health behavior and medical service usage datasets for diverse diseases requiring prescreening. We also expect to use heterogeneous input data such as detailed variables and physiological signals to achieve better prediction performance and to apply the proposed model using a more balanced dataset to reduce the high false-positive rate and improve precision. Finally, we will extend auto-fine-tuning to reduce training time and use a larger-scale method to improve performance.

## Figures and Tables

**Table 1:** AUC results based on the various PCA types.

**Effects of PCA**	**Type**	**Min/Max**	**Quantile Transform**	**Standard**	**PCA without Scaler**	**without PCA**
	AUC	**0.855**	0.788	0.804	0.779	0.695

The top AUC value is highlighted in bold.

**Table 2:** AUC results based on the various hyperparameter setting combination.

Neurons	# of Neuron	10	20	30	40	50
	AUC	0.783	0.783	**0.855**	0.822	0.816
Hidden Layers	# of Layers	2	3	4	5	8
	AUC	0.822	0.832	**0.855**	0.815	0.741
Epochs	Epoch	25	30	40	50	100
	AUC	0.788	0.795	0.814	**0.855**	0.811
Drop-Outs	% of Drop-Out	0.1	0.2	0.3	0.4	0.5
	AUC	0.817	0.821	0.795	**0.855**	0.781

The top AUC values are highlighted in bold.

**Table 3:** Confusion matrix of the proposed model.

Confusion Matrix Parameters	Predicted (Dementia)	Predicted (Non-Dementia)
Actual (Dementia)	23 (TP)	12 (FN)
Actual (Non-Dementia)	455 (FP)	1901 (TN)

**Table 4:** Performance result of the proposed model with min/max scaled PCA.

Classification Model	Threshold	Rc	Sp	Pc	Acc	AUC
DNN	0.025	*68.6*	*82.1*	*5.4*	*81.9*	***85.5***
RF	0.02	65.7	75.3	3.8	75.2	**77.6**
ABC	0.465	62.8	73.7	3.4	73.5	74.1
GNB	0.035	65.7	79.3	4.5	79.1	77.2
MLP	0.005	54.2	79.1	3.7	78.8	75.3
SVC	0.035	65.7	64.5	2.6	64.5	67.6

The top two AUC value are highlighted in bold and DNN results are marked as italic.

**Table 5:** Comparison of performance and methodology.

Methods	# of Subject		# of Features	Performance	Note
	Normal	Dementia			
RF, SVM [37]	40,736	614	4894	AUC (0.775)	-
Logistic Regression with LASSO [36]	16,655	498	EHR	AUC (0.809)	Patients with unrecognized dementia
MLP, SVM [35]	9799	4201	14 for phase 1 31 for phase 2	F-measure (0.739)	High positive cases
PCA/DNN	6928	103	22	AUC (0.855)	-

**Table 6:** Correlation coefficients of the 22 variables.

Variable	Correlation	Variable	Correlation
year	0.003220	arthritis	−0.033356
region	−0.000858	diabetes	−0.004743
age	**0.147275**	hypertension	−0.034224
gender	0.035885	stroke	*0.075265*
marital status	−0.059214	myocardial infarction	−0.004670
education	−0.033166	tuberculosis	−0.013385
insurance type	0.004591	asthma	−0.011954
the number of family members	*0.078062*	chronic renal failure	0.017108
household income	0.018608	smoking status	−0.025947
subjective health status	*0.074173*	drinking	−0.027069
stress awareness	−0.036353	regular exercise	−0.026474

The best correlation (greater than 0.14) is marked in bold and the next three informative values (over ±0.07) are marked in red italics.
